# H_2_O_2_-Driven Anticancer Activity of Mn Porphyrins and the Underlying Molecular Pathways

**DOI:** 10.1155/2021/6653790

**Published:** 2021-03-15

**Authors:** Ines Batinic-Haberle, Artak Tovmasyan, Zhiqing Huang, Weina Duan, Li Du, Sharareh Siamakpour-Reihani, Zhipeng Cao, Huaxin Sheng, Ivan Spasojevic, Angeles Alvarez Secord

**Affiliations:** ^1^Department of Radiation Oncology, Duke University School of Medicine, Durham, NC 27710, USA; ^2^Department of Obstetrics and Gynecology, Division of Reproductive Sciences, Duke Cancer Institute, Duke University School of Medicine, Durham, NC 27710, USA; ^3^Departments of Anesthesiology, Neurobiology, and Neurosurgery, Duke University School of Medicine, Durham, NC 27710, USA; ^4^Department of Medicine, Duke University School of Medicine, Durham, NC 27710, USA; ^5^Pharmacokinetics/Pharmacodynamics (PK/PD) Core Laboratory, Duke Cancer Institute, Duke University School of Medicine, Durham, NC 27710, USA; ^6^Department of Obstetrics and Gynecology, Division of Gynecologic Oncology, Duke Cancer Institute, Duke University School of Medicine, Durham, NC 27710, USA

## Abstract

Mn(III) *ortho*-*N*-alkyl- and *N*-alkoxyalkyl porphyrins (MnPs) were initially developed as superoxide dismutase (SOD) mimics. These compounds were later shown to react with numerous reactive species (such as ONOO^−^, H_2_O_2_, H_2_S, CO_3_^•-^, ascorbate, and GSH). Moreover, the ability of MnPs to oxidatively modify activities of numerous proteins has emerged as their major mechanism of action both in normal and in cancer cells. Among those proteins are transcription factors (NF-*κ*B and Nrf2), mitogen-activated protein kinases, MAPKs, antiapoptotic bcl-2, and endogenous antioxidative defenses. The lead Mn porphyrins, namely, MnTE-2-PyP^5+^ (BMX-010, AEOL10113), MnTnBuOE-2-PyP^5+^ (BMX-001), and MnTnHex-2-PyP^5+^, were tested in numerous injuries of normal tissue and cellular and animal cancer models. The wealth of the data led to the progression of MnTnBuOE-2-PyP^5+^ into four Phase II clinical trials on glioma, head and neck cancer, anal cancer, and multiple brain metastases, while MnTE-2-PyP^5+^ is in Phase II clinical trial on atopic dermatitis and itch.

## 1. Introduction

Besides summarizing the development and bioavailability of MnPs, we aimed herein to review the most recent knowledge on their impact on molecular pathways in normal and cancer cells in the presence of H_2_O_2_. Hydrogen peroxide plays a key role in cellular metabolism as well as in the actions of MnPs. Anticancer effects of MnPs were explored with different sources of H_2_O_2_ in breast, ovarian, and head and neck cancers, glioma, melanoma, and hematological malignancies. Besides radiation and chemotherapy, an additional source of H_2_O_2_—exogenous ascorbate—was recently utilized in breast and ovarian cancers. In the case of ascorbate, MnP cycles with it catalytically, thus giving rise to large amounts of H_2_O_2_. H_2_O_2_ subsequently couples with MnP and GSH to *S*-glutathionylate cysteines of numerous proteins, inactivating them and in turn inducing cancer cell death. The yield of the protein oxidation/*S*-glutathionylation, and therefore the therapeutic efficacy of MnPs, depends on the levels of reactants and their colocalization. MnPs prefer accumulation in cancer relative to normal cell/tissue. Further, cancer is under oxidative stress and has higher levels of H_2_O_2_ than normal cells; such scenario is in the origin of the differential effects of MnPs in inducing cancer cell death while suppressing damage from oxidative insult in a normal cell. We have summarized herein the strategies on the use of different sources of H_2_O_2_, including ascorbate, in breast and ovarian cancers. Ascorbate enhanced the tumor radiosensitizing ability of several cationic MnPs (including MnTnBuOE-2-PyP^5+^) to suppress the viability of 4T1 breast cancer cells and 4T1 tumor growth, due primarily to the modified cellular redox status, massive protein oxidation, and preferred MnP accumulation in cancer cells/tissues. Recent studies on low-grade HOC7 and high-grade CAOV2 serous ovarian cancer cell lines recapitulated such ability of ascorbate to sensitize these cells to MnTnBuOE-2-PyP^5+^. Mouse sc xenograft studies on the CAOV2 cancer cell line confirmed the therapeutic potential of MnTnBuOE-2-PyP^5+^/ascorbate in ovarian cancer.

## 2. Design of Mn Porphyrins as SOD Mimics

Mn(III) substituted pyridylporphyrins (collectively abbreviated as MnPs) were initially developed as powerful mimics of the superoxide dismutase (SOD) family of enzymes [1–4]. The macrocyclic structure of the porphyrin ligand assures the integrity of the metal center where all reactions occurred—the very same reason nature has used the porphyrin ligand for numerous enzymes and proteins such as hemoglobin, myoglobin, cyt P450 family of enzymes, and nitric oxide synthases. The porphyrin ligand itself has redox chemistry outside the range that would have allowed its reactions with biomolecules; moreover, the porphyrin ligand is a powerful photosensitizer. Fe porphyrins were found inferior to Mn analogs for development as indicated at the end of this chapter. Substituted pyridylporphyrins were selected as ligands as they offer favorable thermodynamics and kinetics for the catalysis of O_2_^•-^ dismutation. Structure-activity relationship (SAR) and the ability of MnPs to protect SOD-deficient *Escherichia coli* (*E. coli*) have guided us in their development. The *E. coli* assay has also been used as a major assessment of the toxicity of Mn porphyrins. SAR relates rate constants for the catalysis of superoxide, O_2_^•-^ dismutation, *k*_cat_(O_2_^•−^) to the metal-centered reduction potentials, *E*_1/2_ for the Mn^III^/Mn^II^ redox couple. *E*_1/2_ indicates whether the reaction can happen, while *k*_cat_(O_2_^•−^) indicates how fast the reaction happens. The first compounds that have extraordinarily high *k*_cat_(O_2_^•−^) of ≥7.1 ∙ 10^8^ M^−1^ s^−1^ (approaching that of SOD enzymes, *k*_cat_(O_2_^•−^) ~ 10^9^ M^−1^ s^−1^) were *meta* (3) and *para* (4) isomers of Mn(II) *β*-octabromo *meso*-tetrakis(*N*-methylpyridinium-3,4-yl)porphyrin, Mn^II^Br_8_TM(3 and 4)PyP^4+^. Yet, both compounds have Mn in +2 oxidation states; thus, they are insufficiently stable and readily lose Mn at a biological pH of 7.4 [5]. However, these compounds provided the proof of principle for establishing SAR and developing the powerful and stable porphyrin-based SOD mimics.

**Figure 1:**
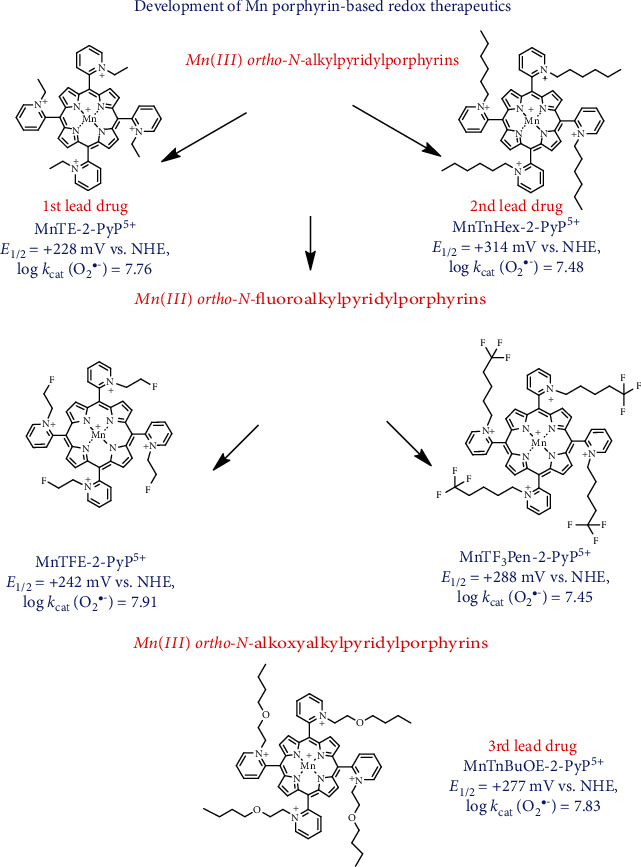
Development of Mn porphyrins towards analogs of optimized efficacy, bioavailability, and safety/toxicity [4, 25]. *Ortho* positions of *N*-pyridyl substituents, due to the vicinity to the Mn site and the cationic charge, afford the best thermodynamics and kinetics for the catalysis of O_2_^•-^ dismutation in an SOD-like fashion; we named the key feature of such design the *ortho* effect. Based on the *ortho* effect, the first generation of powerful SOD mimics was synthesized [6, 26]. Among those, a hydrophilic, MnTE-2-PyP^5+^ (BMX-010), became our 1st lead drug. Its ethylpyridyl chains were subsequently lengthened from up to 8 carbon atoms in order to increase the lipophilicity of the molecule [7]. Among those compounds, MnTnHex-2-PyP^5+^ has the best balance between the lipophilicity (that would not damage cellular membranes at low clinically relevant concentrations) and its SOD-like potency and became our 2nd lead drug. To further diminish the toxicity of MnTnHex-2-PyP^5+^, we introduced the oxygen atoms into hexyl chains deep into the porphyrin cavity. The butoxyethyl analog was created, MnTnBuOE-2-PyP^5+^ (BMX-001), and this became our 3rd lead drug. The approach was similar to the one where ether of sodium dodecyl sulfate (SDS) was synthesized to reduce the toxicity of SDS [10]. Based on the same principles of the *ortho* effect, the *N*-alkylpyridyl groups were fluorinated and two analogs were created: Mn(III) *meso*-tetrakis(*N*-fluoroethylpyridinium-2-yl)porphyrin, MnTFE-2-PyP^5+^ (BMX-002), and Mn(III) *meso*-tetrakis(*N*-trifluoropentylpyridinium-2-yl)porphyrin, MnTF_3_Pen-2-PyP^5+^ (BMX-003). Aqueous chemistry data suggest improved safety/toxicity and efficacy profiles of fluorinated *vs.* nonfluorinated analogs. Initial studies confirmed their potency in radioprotecting normal tissue and radio- and chemosensitizing the 4T1 mouse breast cancer cell line in cellular and animal models [23, 24]. Below each of the structures, the metal-centered reduction potential for the Mn(III)/Mn(II) redox couple and log *k*_cat_(O_2_^•−^) for the catalysis of O_2_^•-^ dismutation are listed.

A very stable and potent SOD mimic, selected based on SAR, was Mn(III) *meso*-tetrakis(*N*-ethylpyridinium-2-yl)porphyrin, MnTE-2-PyP^5+^ (BMX-010, AEOL10113), with *E*_1/2_ = +228 mV*vs.* NHE (normal hydrogen electrode) and log *k*_cat_(O_2_^•−^) = 7.76. This compound became our 1st lead drug from the *first generation of Mn(III) N-alkylpyridylporphyrins* [6] (Figure 1). Its hydrophilicity was thought to be a limiting factor for its distribution within cells and cellular organelles, most so within mitochondrion. A series of alkyl analogs with pyridyl substituents ranging from methyl to n-octyl were then synthesized to improve the lipophilicity and in turn biodistribution of MnPs [7]. Among those compounds, Mn(III) *meso*-tetrakis(*N*-n-hexylpyridinium-2yl)porphyrin MnTnHex-2-PyP^5+^ has balanced redox potency and bioavailability with minimal toxicity to normal cells/tissues [4, 8]; thus, it became our 2nd lead drug with *E*_1/2_ = +314 mV*vs.* NHE and log *k*_cat_(O_2_^•−^) = 7.48 (Figure 1).

Further work was aimed at reducing toxicity to normal cells/tissues, while maintaining high lipophilicity and favorable redox properties. This was achieved by introducing oxygen atoms into *N-*alkylpyridyl chains and led to the creation of the *second generation of Mn(III) N-alkoxyalkylpyridylporphyrins* [9]. The 3rd lead drug emerged from that family of Mn porphyrins—Mn(III) *meso*-tetrakis(*N*-n-butoxyethylpyridinium-2yl)porphyrin MnTnBuOE-2-PyP^5+^ (BMX-001). It has an *E*_1/2_ of +277 mV *vs.* NHE and a log *k*_cat_(O_2_^•−^) of 7.83 (Figure 1) [10]. The synthetic path leading to this compound was cumbersome; its mechanistic aspects were reported in [10, 11]. In addition, a hexoxy analog, Mn(III) *meso*-tetrakis(*N*-n-hexoxyethylpyridinium-2yl)porphyrin, MnTnHexOE-2-PyP^5+^, was synthesized also [12]. For the large part of this review, we have focused on MnTnBuOE-2-PyP^5+^ due to its clinical relevance in cancer therapy. It was shown safe and well tolerated in Phase I clinical trial, where initial data suggest its efficacy [13–15]; it is now in several Phase II clinical trials.

*A third generation of compounds* has been recently developed where *N*-alkylpyridyl substituents were fluorinated (Figure 1). Fluorination of the drugs was known to result in more desirable pharmacokinetic and pharmacodynamic properties due to the increased polarity while at the same time increased lipophilicity and thus bioavailability [16–22]. Fluorine is the most electronegative element in the periodic system of elements. Such nature of fluorine limits our synthetic options when derivatizing porphyrins. Before embarking on the development of *N*-alkoxyalkylpyridylporphyrins, we attempted to replace 5 or 3 hydrogen atoms of MnTE-2-PyP^5+^. Yet, we failed to synthesize a tri- or pentafluorinated ethyl analog, MnTE-2-PyP^5+^. Due to the close proximity, the electronegative fluorines impose overwhelming electron-withdrawing effect on the pyridyl nitrogens. After completing characterization of oxygen-derivatized Mn(III) *N*-alkylpyridylporphyrins, we reassessed the fluorination of MnPs and succeeded in replacing only one hydrogen of MnTE-2-PyP^5+^ in Mn(III) *meso*-tetrakis(*N*-fluoroethylpyridinium-2yl)porphyrin, MnTFE-2-PyP^5+^ (BMX-002). We were then able to replace three hydrogens with fluorines in a pentyl analog as those fluorines were far away from the nitrogen atoms of the *meso* pyridyl rings. Such strategy gave rise to Mn(III) *meso*-tetrakis(*N*-trifluoropentylpyridinium-2yl)porphyrin MnTF_3_Pen-2-PyP^5+^ (BMX-003). As anticipated, both compounds have increased lipophilicity and reduced toxicity relative to nonfluorinated analogs as assessed in an *E. coli* assay [23, 24]. Another example of the reduced toxicity relates to the blood pressure drop, which imposes a limitation to the dosing of MnPs. Both MnTFE-2-PyP^5+^ and MnTF_3_Pen-2-PyP^5+^ exhibit a significantly lower blood pressure drop than MnTnBuOE-2-PyP^5+^ [23].

In combination with ascorbate and radiation, two fluorinated porphyrins were already tested in the 4T1 breast cancer mouse subcutaneous flank model: MnTFE-2-PyP^5+^ (BMX-002) and MnTF_3_Pen-2-PyP^5+^ (BMX-003). Data showed that ascorbate enhances the ability of those compounds to radiosensitize tumor. Also, MnTF_3_Pen-2-PyP^5+^/ascorbate proved efficacious in the radioprotection of prostate and erectile function. Their efficacy is comparable to the efficacy of earlier analogs, which warrants their progression to the clinic [23, 24].

There are a number of Mn complexes, other than *ortho*-*N*-alkyl- or alkoxyalkylpyridyl porphyrins, that have been also explored and discussed in a number of our reviews [2–4, 27, 28]. Among those, the most frequently studied have been Mn(III) corroles [29], Mn(III) salen (EUK) series of compounds [30], anionic Mn(III) porphyrin MnTBAP^3-^ and cationic Mn(III) *meso*-tetrakis(*N*,*N*′-dialkyl- and *N*,*N*′-dimethoxyethyl imidazolium-2-yl)porphyrins, with MnTDE-2-ImP^5+^ (AEOL10150) being the most frequently studied compound. MnTDE-2-ImP^5+^ is also progressing towards the clinic for the radioprotection of normal tissue [9, 31–34]. Several members of the family of Mn cyclic polyamines [35] and Mn complexes whose structures have been inspired by the active site of SOD protein have been also explored [36]. Finally, the Gd and Lu complexes with extended porphyrins—texaphyrins—have been investigated [37]. The anionic Mn(III) *meso*-tetrakis(*p*-carboxylatophenyl)porphyrin, MnTBAP^3-^, is not an SOD mimic. It has negligible catalase and GPx-like activities as well as the ability to oxidize ascorbate and ~300-fold lower ability to reduce peroxynitrite than MnTnBuOE-2-PyP^5+^ and MnTE-2-PyP^5+^ which all account for the therapeutic effects of cationic Mn porphyrins [4, 38]. Though the therapeutic effects of MnTBAP^3-^ were reported by us and others, based on its aqueous chemistry, the origin of such effects cannot be fully understood and were discussed in several of our manuscripts [38–42]. Mn salen compounds have low SOD-like activity, and following activities are negligible: catalase-like activity, ability to oxidize ascorbate and GPx-like activity [43]. Yet in our own hands, in a study on *Cryptococcus neoformans* MnSOD-knockout, EUK-8 was the only compound protective against temperature-induced assault; no other cationic or anionic Mn porphyrin, Tempol, or MnCl_2_ were efficacious [44]. Among the Mn salen EUK series, Mn EUK-134 is in use in humans—in Estee Lauder cosmetic products [30, 43]. The study on *Cryptococcus neoformans* is one of many that clearly indicates that so much is left to comprehend with regard to the interaction of redox-active compounds with components of the redox biology of a cell.

In addition to the Mn porphyrins, their Fe analogs have been extensively studied also [26, 45]. Analogous Fe porphyrins are as powerful SOD mimics as Mn analogs, yet more prone to oxidative degradation [45]. Moreover, they differ with regard to their coordination chemistry [26, 45]. Due to the propensity of the Fe center towards axial coordination, in an aqueous solution Fe porphyrin exists as a monohydroxo/monoaqua species, while Mn porphyrin exists as diaqua species (water molecules are usually not shown). Yet in a rich cellular milieu, Fe porphyrin would readily coordinate biomolecules. This would in turn prevent its Fe center to react with reactive species, precluding therapeutic effects of Fe porphyrins [26, 45]. We have tested MnTnHex-2-PyP^5+^(log *k*_cat_(O_2_^•−^) = 7.48) and (OH)FeTnHex-2-PyP^4+^(log *k*_cat_(O_2_^•−^) = 7.53) in a rodent stroke model [46]. Despite favorable plasma pharmacokinetics of the Fe analog and brain levels as high as those of the Mn analog, only Mn porphyrin was efficacious in a rodent stroke model [46]. In addition, the effects of Fe porphyrins were studied in a SOD-deficient *Escherichia coli* in comparison to Mn analogs [26, 45]. (OH)FeTE-2-PyP^4+^ protects SOD-deficient *E. coli* in a concentration range of 0.01-1 *μ*M, but exhibits toxicity at concentrations higher than 1 *μ*M. Mn analog starts to become efficacious at concentrations higher than 5 *μ*M. Due to different growth patterns, further studies were conducted and it was demonstrated that Fe porphyrin works *via* delivering Fe to the cell [45]. Taken together, our data on Fe porphyrins indicate that more studies are needed before the progress of Fe porphyrins towards the clinic may be considered.

## 3. Bioavailability of Mn Porphyrins

Extensive studies on cationic Mn(III) porphyrins showed their high bioavailability in different cells, cellular organelles, and organs (reviewed in [4]). Their distribution is primarily governed by the positive charges which drive them towards anionic phosphate groups in plasma membrane, mitochondrial membranes, and the nucleus. Their bioavailability is further enhanced by their lipophilicity [4]. The tetracationic Zn analogs were also explored for their biodistribution and were additionally found in lysosomes, membranes, and endoplasmic reticulum [47] indicating that similar distribution may also be true for pentacationic MnPs. The studies on ZnPs demonstrated that the increase in the length of the alkyl chains, and their positions on pyridyl rings affecting the bulkiness/planarity of the molecule, directed the distribution of ZnPs from predominantly lysosomal (ZnTM-2-PyP^4+^) to mitochondrial sites (ZnTnHex-2-PyP^4+^).

MnTE-2-PyP^5+^, MnTnHex-2-PyP^5+^, and MnTnBuOE-2-PyP^5+^ were most closely explored with regard to their bioavailability and pharmacokinetics (PK) (reviewed in [4]). The mitochondrial distribution of MnPs was also explored given the central role of the mitochondria in cellular metabolism under physiological and pathological conditions. MnTE-2-PyP^5+^ was found to preferentially distribute in heart mitochondria relative to cytosol at a 1.6 : 1 ratio [48–51]. Yet, it was only found in brain cytosol, but not in brain mitochondria. The other two lipophilic MnPs prefer heart mitochondria to cytosol at a ratio of ~3 : 1. Moreover, they were found in brain mitochondria *vs.* cytosol at a similar ratio of ~2 : 1 [51, 52]. Such preferential mitochondrial distribution accounts for numerous therapeutic effects of MnPs, many of which are detailed in [27].

All three MnPs are available when given to the mouse subcutaneously (sc), intravenously (iv), intraperitoneally (ip), and topically [4, 28, 49, 53–57]. They have poor oral availability: 0.6, 2.9, and 3.9% for MnTE-2-PyP^5+^, MnTnHex-2-PyP^5+^, and MnTnBuOE-2-PyP^5+^, respectively [4]. Data obtained on MnTnHex-2-PyP^5+^ suggest excellent availability of MnPs if given *via* inhalation [4]. These three MnPs are distributed in all organs, brain included, and to the highest level in the liver and kidney, which serve as depot organs for redistribution in other tissues. The best tissue penetration and retention distribution were found for the most lipophilic MnTnHex-2-PyP^5+^. The initial PK profiles of MnPs were obtained *via* ip and iv routes, at 2 mg/kg for the lipophilic analog MnTnHex-2-PyP^5+^ and at a 5-fold higher dose of 10 mg/kg for the hydrophilic MnTE-2-PyP^5+^. The PK of MnTnHex-2-PyP^5+^ was characterized by higher plasma *t*_max_ (1 h), similar plasma *C*_max_ (1.52 *μ*M), higher plasma *C*_24_ (0.01 *μ*M), slower plasma *t*_1/2_ of elimination (3.8 h), and higher body exposure in many organs, described by the area under the curve (AUC) than MnTE-2-PyP^5+^ (*t*_max_ (0.17 h), *C*_max_ (11.57 *μ*M), *C*_24_ (0.005 *μ*M) and *t*_1/2_ of elimination (6.4 h)) [49, 53, 55]. The plasma AUC_ip_ was 83% of AUC_iv_ for MnTE-2-PyP^5+^ and 84% for MnTnHex-2-PyP^5+^.

The mouse PK of MnTnBuOE-2-PyP^5+^ was done *via* the sc route at 10 mg/kg in comparison with the iv route at 2 mg/kg [28, 54, 56, 57]. The following PK parameters for sc PK were calculated: plasma *t*_max_ (1.3 h), plasma *C*_max_ (6.2 *μ*M), and plasma AUC (23.58 *μ*M·h). The high body exposure was achieved *via* the sc route of administration, which also affords the safest delivery of MnP compared to the iv route with a lesser dose-limiting side effect—blood pressure drop. Again, the highest levels of the drug were found in the liver and kidney. Comprehensive mouse brain PK of MnTnBuOE-2-PyP^5+^ was also performed *via* the sc route in the olfactory bulb, brainstem, cerebellum, thalamus, hippocampus, and cortex [58]. After 10 days of sc administration of 1.5 mg/kg twice daily, the brain levels of lipophilic MnTnBuOE-2-PyP^5+^and MnTnHex-2-PyP^5+^ were between 15 and 160 nM. Much lower levels in brain parts were found for MnTE-2-PyP^5+^ (ranging from 0.8 to 36 nM) [58]. In a dog PK study, MnTnBuOE-2-PyP^5+^ was injected sc for 3 weeks at 0.25 mg/kg three times per week [4, 59]. The highest levels were found in peripheral lymph nodes (ranging from 4 to 6 *μ*M), followed by the levels in the liver (2.5 *μ*M) and kidney (2.0 *μ*M) [4]. Such bioavailability suggests the therapeutic potential of MnTE-2-PyP^5+^ in the treatment of lymphoma [60, 61].

Out of the 3 MnPs, MnTE-2-PyP^5+^ has the safest toxicity profile, but the data demonstrate that MnTnBuOE-2-PyP^5+^ poses no genotoxic risk to humans [55, 56]. Both compounds proved to be safe and well tolerated in Phase I, and both progressed to the Phase II clinical trials [13–15].

## 4. Mn Porphyrins as Redox-Active Compounds

### 4.1. Reactivity of Mn Porphyrins with Low-Molecular-Weight Reactive Species

While initially developed as SOD mimics, MnPs do not possess the quaternary structure of an enzyme to be specific for the reaction with superoxide (O_2_^•-^). MnPs are small molecules with no steric hindrance towards incoming reactive species. Indeed, they are almost as reactive with peroxynitrite (ONOO^−^), as with O_2_^•-^ [62]. Our insight into their reactivities matured along with mounting knowledge on the redox biology. The very first insight into MnPs' reactivity, other than towards O_2_^•-^, was provided in collaboration with Radi et al.; the reactions towards peroxynitrite (ONOO^−^) and carbonate radical (CO_3_^•-^) were explored [63, 64]. A decade later, the same collaboration showed a high activity of MnPs towards hypochlorite, (ClO^−^) [65]. In essence, MnPs are reactive with any reactive species we have thus far explored which in addition to ONOO^−^ and O_2_^•-^ include hydrogen peroxide (H_2_O_2_), ClO^−^, nitric oxide (^•^NO), glutathione (GSH), ascorbate, hydrogen sulfide (H_2_S) [66, 67], oxygen (O_2_), sulfite (SO_3_^2-^), tetrahydrobiopterin (BH_4_), and protein (cysteine) thiols (R-SH) [4, 62]. Due to the complex chemistry of MnPs with 4 oxidation states available for *in vivo* reactions, and complex biological milieu, many more reactions are to be uncovered. The important message from studies in aqueous milieu is that the more potent the SOD mimic Mn porphyrin is, the more potent it is also in undergoing redox reactions with reactive species thus far explored other than O_2_^•-^, namely, ONOO^−^, ClO^−^, H_2_S, ascorbate, H_2_O_2_, and thiols via the GPx-like reactivity [4, 48, 49, 65–67]. Numerous efficacy studies further demonstrated that the more potent the SOD mimic Mn porphyrin is, the more efficacious therapeutic it tends to be [2–4, 62, 68, 69].

The critical role of the SOD enzyme in tumorigenesis staged grounds for the exploration of SOD mimics in cancer [27, 70, 71]. We have early on demonstrated that MnPs inhibit transcription factors, such as hypoxia-inducible factor (HIF-1*α*), activator protein (AP-1), and specificity protein (SP-1) [3, 4, 62]. We had no clue, until more than 10 years later, what was really happening, *i.e.*, how a small molecule such as MnP interacts with a huge protein structure and in the process inhibits its activity. The first explanation was that MnP removes reactive species, such as O_2_^•-^ and ONOO^−^ (in antioxidative fashion), which would have otherwise served as a signal for the activation of proteins involved in apoptotic/proliferative pathways. We knew early on that MnP reacts with endogenous small-molecular-weight antioxidants, oxidizing ascorbate one-electronically into ascorbyl radical [26] and glutathione into glutathione radical [48]. We have overlooked the seemingly obvious fact that during the dismutation process, MnP does act as a prooxidant in the 1st step of the process, oxidizing O_2_^•-^ into oxygen. Despite such evidence, we have not for a long while considered that the prooxidative action of MnP happens *in vivo* and may play a major role in the overall mechanism of action of MnPs. The very first suggestion of a prooxidative action of MnP from *in vivo* studies came from our colleague Jon Piganelli; he suggested that MnTE-2-PyP^5+^ oxidizes the p50 subunit of nuclear factor-*κ*B (NF-*κ*B) in the nucleus, preventing its transcription [72]. The subsequent collaborative study demonstrated the feasibility of such notion due to ~3-fold higher accumulation of MnTE-2-PyP^5+^ in the nucleus than in cytosol [48]. Still, at that point in time, we did not understand the type of reaction/s behind such event.

### 4.2. Reactivity of Mn Porphyrins towards Protein Cysteines -Thiol Signaling

Independent studies by Tome's lab were crucial to understand the nature of the major action/s of MnPs *in vivo*, and in particular in *cancer cells* [60, 61, 73]. Her studies on the lymphoma cellular model showed that MnTE-2-PyP^5+^ alone (cycling with cellular reductants such as ascorbate as described in equations (1)–(3)), and more so in the presence of dexamethasone, increases the levels of H_2_O_2_ [74]. In the next step, MnP employs H_2_O_2_ to undergo oxidation into high-valent and highly oxidizing Mn(IV) [starting from reduced Mn(II)P, not shown] and Mn(V) oxo species [starting from Mn(III)P, equation (4)]. These species [shown for Mn(III)P] can subsequently oxidize the protein cysteine residue, Prot-Cys-S^−^, into the Prot-Cys-S^•^ radical (equation (5)). The Prot-Cys-S^•^ radical then couples with another radical to make disulfide. Disulfide exchanges glutathione in the presence of GSH (equation (6)) and gets *S-*glutathionylated (equation (7)) [60, 61]. The high-oxo MnP gets recycled back into its resting state, Mn^III^P^5+^, with a cellular reductant such as ascorbate and glutathione (equations (8) and (9)). The kinetics of the reaction of O = Mn^IV^TM − (2, 3 and 4)PyP^4+^ with tyrosine, glutathione, and ascorbate was reported by Radi's team [63]. High-valent MnPs can also oxidize GSH giving rise to GSSG (equation (10)). Several reactions, in addition to those shown below, can also account for the protein *S*-glutathionylation (see other possibilities in [4, 43, 74]). In one of those scenarios, instead of reacting with another Pr(Cys)-S^•^ radical to form disulfide (equation (6)), Pr(Cys)-S^•^ reacts with GSH. Such reaction results in the formation of a Prot(Cys)-S-S-G^•-^ anion radical, which then reacts with oxygen giving rise to glutathionylated protein, Prot(Cys)-S-S-G [43]. The high-valent Mn(V) dioxo species can also oxidize its own porphyrin ligand giving rise to the Mn(IV) porphyrin *π*-cation radical, O = Mn(IV)P^•+^, which would result in the degradation of the Mn complex [75]. The involvement of H_2_S/MnP in the *S*-glutathionylation of proteins was also implicated [66, 67]. In the study of Tome's lab, MnP oxidizes primarily cys38 of the p65 subunit of NF-*κ*B and to a lesser degree cys62 of the p50 subunit. Her team provided unambiguous evidence that H_2_O_2_ and GSH are necessary for such protein oxidative modification to occur. The team showed that no *S*-glutathionylation happened when catalase was overexpressed or GHS synthesis inhibited [74]. Finally, Tome's team provided direct evidence that *S*-glutathionylation was the type of oxidative damage to NF-*κ*B inflicted by MnP [61]. Such work helped us understand how the small Mn porphyrin molecule reacts with cysteines exposed at the surface of proteins, and in turn affects the cellular signaling pathways.

In addition to NF-*κ*B, Tome's lab showed that complexes I, III, and IV of the mitochondrial respiration were also oxidized by MnP/H_2_O_2_/GSH. Complexes I and III, but not complex IV, were inactivated by such oxidative modification. Data suggest that some proteins involved in glycolysis may also be oxidized by the action of MnP/H_2_O_2_/GSH [60]. Importantly, MnP did not oxidize proteins in normal lymphocytes [60].

In addition to the glucocorticoid, dexamethasone, Tome's team also demonstrated that MnPs have potential for use in hematologic malignancies that are treated with cyclophosphamide and doxorubicin [73]. While MnTE-2-PyP^5+^ sensitized murine thymic lymphoma cells to cyclophosphamide, it protected H9c2 cardiomyocytes from doxorubicin toxicity [73].

Jones referred to *S*-glutathionylation as a major *in vivo* thiol oxidative modification [76, 77]. MnP gets involved in thiol signaling, *via* reversible *S*-glutathionylation, providing an on/off switch for an individual protein. More details on the proteins, which activities are thus far found modified by MnPs, are listed below under “Molecular Pathways Affected by Mn Porphyrins in Cancer and Normal Cell” :

(1) $$\begin{array}{c} {\text{Mn}}^{\text{III}}{\text{P}}^{5+}+{\text{HAsc}}^{-}⟺{\text{Mn}}^{\text{II}}{\text{P}}^{4+}+{\text{HAsc}}^{•},\;\text{reduction with ascorbate} \end{array}$$

(2) $$\begin{array}{c} \text{M}{\text{n}}^{\text{II}}{\text{P}}^{4+}+{\text{O}}_{2}⟺\text{M}{\text{n}}^{\text{III}}{\text{P}}^{5+}+{\text{O}}_{2}^{•-},\;\text{oxidation with oxygen} \end{array}$$

(3) $$\begin{array}{c} 2{\text{O}}_{2}^{•-}+2{\text{H}}^{+}⟺{\text{H}}_{2}{\text{O}}_{2}+{\text{O}}_{2},\;\text{production of }{\text{H}}_{2}{\text{O}}_{2} \end{array}$$

(4) $$\begin{array}{c} {\text{Mn}}^{\text{III}}{\text{P}}^{5+}+{\text{H}}_{2}{\text{O}}_{2}⟺{\left(\text{O}\right)}_{2}{\text{Mn}}^{\text{V}}{\text{P}}^{3+}+2{\text{H}}^{+},\;\text{oxidation of M}{\text{n}}^{\text{III}}\;{\text{P}}^{5+}\text{ with }{\text{H}}_{2}{\text{O}}_{2} \end{array}$$

(5) $$\begin{array}{c} {\left(\text{O}\right)}_{2}{\text{Mn}}^{\text{V}}{\text{P}}^{3+}+2{\text{H}}^{+}+\text{Prot}\left(\text{Cys}\right)-{\text{S}}^{-}⟺\text{O}={\text{Mn}}^{\text{IV}}{\text{P}}^{4+}+\text{Prot}\left(\text{Cys}\right)-{\text{S}}^{•}+{\text{H}}_{2}\text{O},\;\text{oxidation of protein cysteine} \end{array}$$

(6) $$\begin{array}{c} \text{Prot}\left(\text{Cys}\right)-{\text{S}}^{•}+\text{Prot}\left(\text{Cys}\right)-{\text{S}}^{•}⟺\text{Prot}\left(\text{Cys}\right)-\text{S}-\text{S}-\left(\text{Cys}\right)\text{Prot},\;\text{disulfide formation} \end{array}$$

(7) $$\begin{array}{c} \text{Prot}\left(\text{Cys}\right)-\text{S}-\text{S}-\left(\text{Cys}\right)\text{Prot}+\text{GSH}⟺\text{Prot}\left(\text{Cys}\right)-\text{S}-\text{S}-\text{G}+\text{Prot}\left(\text{Cys}\right)-\text{SH},\;\text{glutathione exchange} \end{array}$$

(8) $$\begin{array}{c} \text{O}={\text{Mn}}^{\text{IV}}{\text{P}}^{4+}+2{\text{H}}^{+}+{\text{HAsc}}^{-}⟺\text{M}{\text{n}}^{\text{III}}\;{\text{P}}^{5+}+{\text{HAsc}}^{•}+{\text{H}}_{2}\text{O}\left({\text{HAsc}}^{•}⟺{\text{Asc}}^{•-}+{\text{H}}^{+}\right),\;\text{Mn porphyrin regeneration} \end{array}$$

(9) $$\begin{array}{c} \text{O}={\text{Mn}}^{\text{IV}}{\text{P}}^{4+}+2{\text{H}}^{+}+{\text{GS}}^{-}⟺\text{M}{\text{n}}^{\text{III}}\;{\text{P}}^{5+}+{\text{GS}}^{•}+{\text{H}}_{2}\text{O},\;\text{Mn porphyrin regeneration} \end{array}$$

(10) $$\begin{array}{c} {\left(\text{O}\right)}_{2}{\text{Mn}}^{\text{V}}{\text{P}}^{3+}+2{\text{GS}}^{-}+4{\text{H}}^{+}⟺\text{M}{\text{n}}^{\text{III}}\;{\text{P}}^{5+}+\text{GSSG}+2{\text{H}}_{2}\text{O},\;\text{glutathione oxidation} \end{array}$$

MnP does not discriminate cancer cells from normal cells. Thus, the same type of reactions would occur in both normal and cancer cells, but at different yields controlled by much higher levels of H_2_O_2_ and MnP in cancer cells than in normal cells [43]. High yields of oxidation of proteins in cancer, such as NF-*κ*B and mitogen-activated protein kinases (MAPKs) would subsequently result in massive apoptosis of cancer cells (see below under “Molecular Pathways Affected by Mn Porphyrins in Cancer and Normal Cell”).

In a *normal cell*, H_2_O_2_ (endogenously formed in many processes) is, however, kept at nM level with abundant enzymes, such as catalase, glutathione peroxidase, and peroxiredoxins and their partner proteins, thioredoxins. Under an oxidative insult, MnP^5+^ would cycle in the cell with cellular reductants such as ascorbate (equations (1)–(3)) and glutathione or protein cysteines and would be reduced to Mn^II^P^4+^, which would then be reoxidized to Mn^III^P^5+^, thereby reducing oxygen to O_2_^•-^. O_2_^•-^ would in turn enzymatically or spontaneously dismute to H_2_O_2_ and O_2_ (equation (3)). The production of H_2_O_2_, while not excessive in a normal cell, would just be sufficient to be employed by MnP for the catalysis of oxidation and inhibition of NF-*κ*B, oxidation of Kelch-like ECH-associated protein 1 (Keap1), and activation of nuclear factor E2-related factor 2 (Nrf2) [4]. Moderate inhibition of NF-*κ*B would in turn suppress exccessive inflammatory responses. The activation of Nrf2 would result in the upregulation of cellular endogenous antioxidant defenses including MnSOD, catalase, and glutathione-*S*-transferase that would remove O_2_^•-^ and H_2_O_2_. Thus, in a normal but diseased tissue/cell, modest inhibition of NF-*κ*B and activation of Nrf2 would jointly restore the cellular physiological redox environment.

## 5. Molecular Pathways Affected by Mn Porphyrins in Cancer and Normal Cell

**Figure 2:**
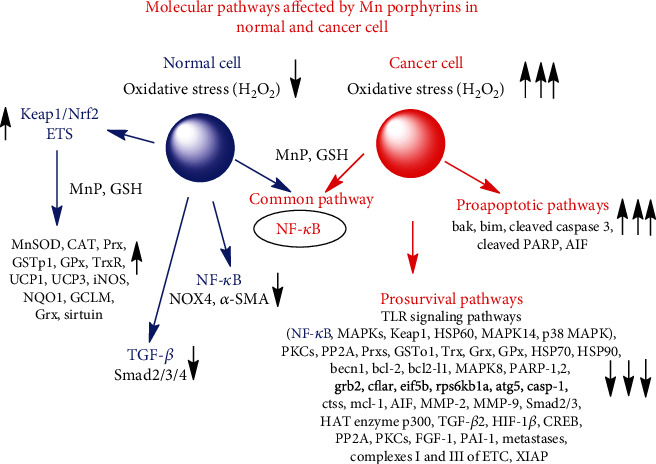
Molecular pathways affected by Mn porphyrins in normal and cancer cell. Most of the data were derived from breast cancer and glioma studies. Two major pathways, affected by Mn porphyrins, have been explored: nuclear factor-*κ*B (NF-*κ*B) and Keap1/nuclear factor E2-related factor 2 (Nrf2). In both tumor and normal tissue injury, NF-*κ*B was inactivated by the MnP-driven catalysis of p50 and p65 cysteine oxidation in the presence of H_2_O_2_ and GSH. The yield of inactivation depends upon MnP and H_2_O_2_ levels, both of which are higher in tumor than in normal cell *Normal cell*. *Hematopoietic stem/progenitor cells*. The nuclear factor E2-related factor 2 (Nrf2) pathway was activated, and it upregulated endogenous antioxidative defenses such as MnSOD, catalase (CAT), glutathione peroxidase (GPx), peroxiredoxin (Prx), glutaredoxin (Grx), glutathione-*S*-transferase (GSTp1), NAD(P)H quinone dehydrogenase (NQO1), uncoupling proteins 1 and 3 (UCP1 and UCP3), and glutamate cysteine ligase regulatory subunit (GCLM). In the study, the ETS transcription activity, which plays role in hematopoiesis, was also increased. The ETS family of transcription factors is among the end effector molecules of the signal transduction pathway. Phosphorylation of ETS modulates DNA binding, protein-protein interaction, transcriptional activation, and subcellular localization. ETS stands for E26 transformation specific or E26, as the ETS sequence (v-ets) was first identified in the genome of the avian retrovirus E26. *Normal colorectal and prostate fibroblasts*. The effect of MnP on NADPH oxidase 4 (NOX4) and *α*-smooth muscle actin (*α*-SMA), Nrf2, NF-*κ*B, and TGF-*β* molecular with a subsequent effect on Smad 2/3 was reported. Smad 2/3 is the main signal transducer for receptors of the transforming growth factor beta (TGF-*β*) superfamily. The abbreviation stands for an acronym from the fusion of *Caenorhabditis elegans* Sma and the *Drosophila* Mad (Mothers against decapentaplegic) genes. *Cancer. 4T1 breast cancer*. NF-*κ*B, a part of the TLR molecular pathway, is the major pathway affected by MnP/ascorbate in 4T1 cells as found by redox proteomics. In addition, and as a part of the TLR pathway, MnP/ascorbate oxidized cysteines of different mitogen-activated protein kinases (MAPKs) (p38 mitogen-activated protein kinase (p38 MAPK) and MAPK14) and protein kinase C (PKC *ι* and *δ*). We have also seen the oxidation of Kelch-like ECH-associated protein 1 (Keap1) as part of the TLR pathway. In addition, endogenous antioxidative defenses were oxidized, such as Cu,ZnSOD, glutathione-*S*-transferase (GSTo1), peroxiredoxins (Prxs), thioredoxin, thioredoxin 1 (Trx1), and heat shock proteins (HSPs). In another 4T1 breast cancer study, the inactivation of c-Jun N-terminal kinase (JNK), extracellular signal-regulated kinase (ERK), and protein kinase B (AKT) by MnP/RT was seen. Also, the suppression of antiapoptotic NF-*κ*B, bcl-2, and mcl-1; the promotion of proapoptotic pathways, bak and bim; and the increase of cleaved PARP and cleaved caspase 3 were demonstrated. *Inflammatory breast cancer*. In SUM149 cells, the inhibitory effect of MnTE-2-PyP^5+^ on NF-*κ*B, p38 MAPK, ERK1/2, XIAP (X-linked inhibitor of apoptosis protein); the activation of apoptosis-inducing factor (AIF); and the increase in PARP cleavage were seen. *Glioblastoma multiforme/glioma*. In glioma tissue from a subcutaneous xenograft mouse study, the cell death array showed a number of genes downregulated by MnP/RT (fold difference *vs.* RT alone listed in parenthesis). Among the most affected genes are those that regulate the following proteins (listed from most affected to less affected): eif5b, eukaryotic translation initiation factor 5B (-408.8-fold); rps6kb1a, serine/threonine kinase that acts downstream of PIP3 (phosphatidylinositol (3,4,5)-trisphosphate) and phosphoinositide-dependent kinase-1 in the PI3 kinase pathway (-176.6-fold); bcl2-l1, a potent inhibitor of apoptosis (-59-fold); ctss, cathepsin S (-59-fold); grb2, growth receptor bound protein-2, an adaptor protein involved in signal transduction/cell communication (-45.5-fold); atg5, autophagy-related protein (-43-fold); cflar, CFLAR, CASP8, and FADD-like apoptosis regulator (-38.7-fold); casp1, inflammasome mediates activation of caspase-1 which promotes the secretion of the proinflammatory cytokines IL-1*β*, IL-18, and pyroptosis (-37-fold); CD40, cluster of differentiation 40, member of the TNF superfamily essential to a broad array of immune and inflammatory responses (-21-fold); becn1, plays critical role in autophagy and cell death (-16-fold); NF-*κ*B p105 subunit (-13.6-fold); MAPK8 (also known as JNK1), mitogen-activated kinase which plays a key role in T cell proliferation, apoptosis, and differentiation (-7.8-fold); PARP-1 and PARP-2, poly(ADP-ribose) polymerase-1 and 2 (-5.4-fold and -3.3-fold, respectively). *Prostate cancer*. The effect of MnP was seen on histone acetyltransferase p300 (HAT p300), cAMP response element-binding protein (CREB), fibroblast growth factor (FGF), hypoxia-inducible factor 1*β*(HIF-1*β*), and plasminogen activator inhibitor-1 (PAI-1). *Colorectal cancer*. MnP-driven suppression of transforming growth factor (TGF-*β*) and in turn matrix-metalloproteinases 2 and 9 (MMP-2 and MMP-9) and Smad 2/3 was reported. *Hematologic malignancies*. NF-*κ*B and complexes I, III, and IV of the mitochondrial electron-transport chain (ETC) were oxidized by MnP.

The MnP-driven oxidation of NF-*κ*B was explored in both normal cell/tissue [72, 78, 79] and cancer and seems to be at least one of the major pathways contributing to the therapeutic effects of MnPs [4, 60, 61, 80, 81]. Yet, the impact on Nrf2 was thus far only explored in normal tissue [82]. Several other pathways affected by MnPs were also explored in cancer but not in normal cells, and were detailed below and listed in Figure 2. Abbreviations of signaling proteins affected by MnPs are described in the legend of Figure 2.

### 5.1. Cancer Cell

The redox proteomics of the *mouse breast 4T1 cancer cell line*, exposed to excessive levels of H_2_O_2_ produced *via* cycling of MnTE-2-PyP^5+^ with ascorbate (equations (1)–(3)), demonstrated that 3605 peptidyl cysteines (Cys) were affected in total, out of which 1577 were oxidized 1.3-fold or higher compared with control, untreated 4T1 breast cancer cells [80]. While cancer cells may be under oxidative stress and have Nrf2 already activated, our data provide clear evidence that the MnP/Asc system oxidized Keap1 which may lead us to assume that this would activate the Nrf2 pathway. The MnP/Asc system affected 1762 proteins in total, among which 942 proteins were associated with >1.3-fold oxidized peptidyl Cys. Such massive oxidative modification of proteins, driven by MnP/ascorbate, promoted apoptotic pathways in tumor cells. The pathways affected were those associated with the regulation of cytoskeleton rearrangement, transcription-mRNA processing, translation, protein folding, cell cycle, and adhesion. Most affected was the toll-like receptor signaling (TLR) with the largest number of pathways involved, the components of which are listed below. The fold oxidation, imposed by MnP/ascorbate relative to nontreated 4T1 cells, is given in parenthesis: NF-*κ*B (1.39-fold), Keap1 (3.73-fold), p38 MAPK (1.36-fold), p38a (MAPK14) (6.84-fold), and heat shock protein HSP60 (4.26-fold). The protein kinase PKC*σ* (1.39-fold) and PKC*ι* (2.74-fold) as well as serine/threonine protein phosphatase 2A subunit A (1.51-fold) have a critical impact on the TLR pathway [80, 83–85]. In addition to HSP60, several other HSPs were found oxidized also. Of our immediate interest, redox proteomics indicated that cysteines of numerous endogenous antioxidative defenses were oxidized, such as glutaredoxin 3 (1.85-fold) and glutaredoxin 5 (2.62-fold); glutathione-*S-*transferase o1 (1.64-fold); isocitrate dehydrogenases 1 (1.87-fold), 2 (1.68), and 3 (2.46-fold); peroxiredoxins 4 (2.26-fold), 5 (1.99-fold), and 6 (1.47-fold); thioredoxin 1 (1.72-fold), and thioredoxin domain containing proteins 5 (1.36-fold) and 6 (2.46-fold). Based on the results from Tome's lab (see under "Reactivity of Mn Porphyrins towards Protein Cysteine-Thiol Signaling") and our data from the mouse breast cancer study (see under "Breast Cancer"), we may safely assume that S-glutathionylation is the type of oxidative modification. Reportedly, such oxidative modification results in protein inactivation [61, 74, 86–90]. In addition, Cu,ZnSOD and catalase were oxidized also.

In a collaborative mouse study with Park's team's a lipophilic analog with similar redox properties , MnTnHex-2-PyP^5+^, radiosensitized 4T1 breast cancer cell line resulting in a large suppression of tumor growth. In accompanying cellular study, MnTnHex-2-PyP^5+^ suppressed the antiapoptotic pathways NF-*κ*B, bcl-2, and mcl-1, while promoting the proapoptotic pathways bim, bak, cleaved PARP, and cleaved caspase 3 (Figure 2) [91]. The authors also found that the radiation-induced activation of several MAPKs (ERK, JNK, AKT and p38 MAPK), was greatly suppresed by MnP; such data are in agreement with the study on redox proteomics of a 4T1 breast cancer cell line exposed to MnP/ascorbate. Finally, the authors reported also the radiosensitization of the B16 melanoma cell line by MnTnHex-2-PyP^5+^ in a mouse model [91].

The effect of MnTE-2-PyP^5+^/ascorbate upon NF-*κ*B, ERK1/2, and p38 MAPK was also seen in a study of the rare and highly aggressive inflammatory breast cancer (IBC) cell line SUM149 (basal type) [92]. MnTE-2-PyP^5+^ significantly depleted glutathione levels and reduced phosphorylated NF-*κ*B, ERK1/2, and p38 MAPK. MnTE-2-PyP^5+^/ascorbate induced the release of the apoptosis inducible factor, AIF, from mitochondria which was followed by its translocation into the nucleus. This event is known to induce caspase-independent cell death. The decreased X-linked inhibitor of apoptosis protein, XIAP, and the increase in cleaved PARP by MnTE-2-PyP^5+^/ascorbate was also demonstrated, revealing apoptosis-mediated cytotoxicity in cells [69]. Both MnTE-2-PyP^5+^ and MnTnBuOE-2-PyP^5+^ combined with ascorbate suppressed the viability of the SUM190 (erb-b2, receptor tyrosine kinase 2, overexpressor cell line), SUM149, and even rSUM149 cell lines. The rSUM149 cell line is an isogenic derivative of SUM149 that shows significant resistance to reactive species-mediated apoptosis due to high levels of GSH, Cu,ZnSOD, and MnSOD.

The effect of MnTnBuOE-2-PyP^5+^, MnTE-2-PyP^5+^, and MnTnHex-2-PyP^5+^ was also explored in studies on *hematologic malignancies* (lymphoma, multiple myeloma, and the activated B cell subtype of diffuse large B cell lymphoma) (see under “Reactivity of Mn Porphyrins towards Protein Cysteine-Thiol Signaling” and Figure 2) [4, 60, 61, 91].

The tumor radio- and chemosensitization was demonstrated in a mouse subcutaneous 245-MG *glioblastoma multiforme* xenograft model when mice were treated with radiation, temozolomide, and either MnTnBuOE-2-PyP^5+^ or MnTnHex-2-PyP^5+^ [3, 93]. A cell death gene array demonstrated a significant decrease in gene expression in tumors of mice treated with MnTnBuOE-2-PyP^5+^/radiation in comparison to radiation only [3, 94]. The metastatic pathways (*ctss* (-58-fold *beclin1*)) were largely downregulated, as were the antiapoptotic and NF-*κ*B pathways (*Bcl2-l1* (-59-fold), *mcl-1* (-8.7-fold), *Nfkb1* (-13.6-fold), and PI3kinase and mTOR (*Rsp6kb1* (-177-fold)); fold decrease is listed in parentheses next to the name of the gene. The protein translation changes were also implicated as *Eif5b* was downregulated by -408.8-fold (Figure 2).

The impact of MnTnBuOE-2-PyP^5+^ combined with radiation was demonstrated in the suppression of the *FaDu human epithelial cell line from squamous cell carcinoma* of the hypopharynx in a mouse sc flank xenograft model. The tumor radiosensitizing effect of MnP was discussed with regard to the significant influx of tumoricidal M1 tumor-associated macrophages [54, 95]. Further, MnTnBuOE-2-PyP^5+^ reduced radiation-mediated mucositis, xerostomia, and fibrosis of salivary glands.

*In prostate cancer* studies, post radiation, MnTE-2-PyP^5+^ significantly reduced the overall HAT activity altering the p300 DNA binding, which resulted in the inhibition of HIF-1*β* and CREB signaling pathways as the expression of 3 of their genes, namely, TGF-*β*, FGF-1, and PAI-1, was reduced [96]. The HAT enzyme, p300, acetylates histones and increases overall transcription. The authors speculate that Mn porphyrin may not only be altering activity of transcription factors directly but may also be affecting transcription globally. Such effect may occur *via* modification of the redox transcription factor enhancer, p300 [96]. In another prostate cancer study, authors speculate that the anticancer effect of MnTE-2-PyP^5+^ on growth inhibition may occur through protein oxidative modifications and mitotic catastrophes in the PC3 prostate cancer cell line, while it may occur through tumor cell quiescence or cell death in the LNCaP prostate cancer cell line [97].

The impact of MnPs on metastases was reported by Oberley-Deegan et al. on colorectal cancer and Fernandes et al. on breast cancer (see below) [98]. *In colorectal cancer*, MnTE-2-PyP^5+^ significantly reduced the expression of mesenchymal markers, suppressing the phosphorylated Smad2/3 protein levels induced by TGF-*β* in SW480 cells. It also suppressed the TGF-*β*-mediated cell migration and invasion and the expression of matrix metalloproteinase 2 (MMP-2) and MMP-9 in colorectal cells [99, 100].

*The human breast cancer cell lines, MCF-7 and MDA-MB-231*, were studied by Flórido et al. [98]. Both cells are invasive ductal/breast carcinomas. While MDA-MB-231 is triple negative to estrogen and progesterone receptor and does not have HER2 amplification (human epidermal growth factor receptor 2), it is multidrug resistant and present in about 15% to 20% of all breast cancer cases. MCF-7 is an estrogen and progesterone receptor-positive cell line. MnTnHex-2-PyP^5+^ enhanced the cytotoxicity of doxorubicin to those breast cancer cells, *via* increasing H_2_O_2_ levels and reducing collective cell migration and chemotaxis thereby affecting their metastatic potential [98]. The reduction in a doxorubicin-induced increase in random migration and proteolytic invasion of MDA-MB-231 cells by MnP was also seen [98]. Breast cancer patients treated with doxorubicin, cyclophosphamide, and paclitaxel frequently exhibit brain toxicity, often described by cancer survivors as chemo brain. In a mouse study, McElroy et al. showed that MnTnBuOE-2-PyP^5+^ ameliorates effects on mature dendrite morphology and neurocognition [101]. The proteomic analysis suggested mitochondrial dysfunction, oxidative stress, and energy metabolism as possible mechanisms behind chemo brain. The most affected pathways by MnP/chemotherapy *vs.* chemotherapy include 3-phosphoinositide degradation, the super pathway of inositol phosphate compounds, the mitochondrial L-carnitine shuttle pathway, polyamine regulation, and Sirtuin signaling. The top network has functions associated with the cardiovascular system and cell morphology.

Driven by H_2_O_2_, MnP also has a beneficial impact on reducing cell viability and chemotactic migration of *human clear renal cancer cells* [102].

### 5.2. Normal Cell

The very first evidence of the possible involvement of the Nrf2 pathway in the therapeutic effects of metalloporphyrins was reported by Konorev et al. [103] in a study on doxorubicin-induced toxicity to cardiomyocytes. One of the genes controlled by Nrf2 is heme oxygenase-1. Konorev et al. demonstrated that anionic metalloporphyrins, (OH)FeTBAP^4-^ and CoTBAP^3-^, induced heme oxygenase-1 and protected cardiomyocytes from DOX-induced apoptosis [103]. MnTBAP^3-^ and ZnTBAP^4-^ were less efficacious. The reasoning behind the SOD-independent (but H_2_O_2_-dependent) suppression of DOX-induced toxicity to cardiomyocytes by Fe and Co porphyrins is as follows. (OH)FeTE-2-PyP^4+^ is ~13-fold better catalase mimic than its Mn analog, MnTE-2-PyP^5+^ [104]. Based on such analogy, and with MnTBAP^3-^ having *k*_cat_(H_2_O_2_) = 5.84, the (OH)FeTBAP^4-^ would have *k*_cat_(H_2_O_2_) of ~76, slightly higher than the *k*_cat_(H_2_O_2_) of MnTE-2-PyP^5+^, which is 63.32; our data show that the latter value allows for the efficient cycling of MnP with H2O2. This in turn implies that (OH)FeTBAP^4-^ could have been oxidized with H_2_O_2_ to the (O_2_)Fe(V) species in the 1st step of the H_2_O_2_ dismutation process. This species of high oxidizing power could have in turn oxidized Keap1 and activated Nrf2.

In a *kidney ischemia/reperfusion model*, rats were treated with the cocktail containing MnTnHex-2-PyP^5+^. 100 *μ*g/kg/day of Mn porphyrin was given at -24, 0, and +24 h after clamping the left renal artery. Ischemia lasted 40 min, followed by 48 h of reperfusion. In comparison to nontreated rats, the real-time quantitative polymerase chain reaction for oxidative stress-related genes showed that numerous antioxidative enzymes were upregulated, such as MnSOD (10.2-fold); extracellular SOD (ECSOD) (12.3-fold); peroxiredoxins 2 (9.6-fold), 3 (9.8-fold), and 5 (6.6-fold); GPx 1 (9.8-fold) and 3 (23.9-fold); and thioredoxin reductase 1 (6.9-fold) [105]. Such data suggested the role of Nrf2 in the protective mechanism of action of Mn porphyrin. This was the first study to suggest that MnP does not act as a SOD mimic in vivo, but rather upregulates the SOD enzyme.

Subsequently, St. Clair et al. provided the first direct evidence on the MnP-driven activation of the Nrf2 pathway. In a study on *hematopoietic stem/progenitor cells (HSPC)*, MnTnBuOE-2-PyP^5+^ enhanced the number and function of those cells under physiological conditions and improved the function of long-term engraftment and multilineage differentiation of HSC. *Via* activation of Nrf2/ARE (antioxidant response element) and ETS transcription activities, MnTnBuOE-2-PyP^5+^ regulated intracellular redox homeostasis, supporting quiescence and possibly affecting bone marrow HSPC regulation. The ETS family of transcription factors, comprising more than 26 genes in vertebrates, have been implicated in the regulation of hematopoiesis. Reduction in Nrf2 activity, however, was shown to enhance the differentiation of hematopoietic stem cells, while the reversal of Nrf2 knockdown increases pluripotency [106]. The upregulation of the following Nrf2-controlled genes was demonstrated by the action of MnP: MnSOD, catalase, glutathione-*S*-transferase p1 (GSTp1), NAD(P)H quinone dehydrogenase (NQO1), uncoupling proteins 1 and 3, and glutamate cysteine ligase regulatory subunit. Finally, the increased expression of MnSOD, catalase, GSTp1, and UCP3 proteins was demonstrated [82]. The data clearly demonstrate that MnP does not act as an SOD mimic but upregulates the SOD enzyme.

The MnP-driven activation of Nrf2 (with upregulation of MnSOD, NQO1, and sirtuin) and the inhibition of NF-*κ*B (accompanied by suppression of NOX4 and *α*-SMA) was subsequently reported by Oberley-Deegan et al. to play a critical role in the radioprotection of *prostate* [107–109]. Further, her team demonstrated that the TGF-*β* pathway also plays a role in MnP-mediated protection of prostate fibroblasts from radiation-induced damage. Thus, MnTE-2-PyP^5+^ downregulates the expression of TGF-*β* receptor 2, with subsequent reduction in activation and/or expression of Smad2, Smad3, and Smad4. Consequently, Smad2/3-mediated transcription of profibrotic markers was reduced [108].

The impact of MnPs on the Keap1/Nrf2 pathway in cancer has not yet been studied. However, in the redox proteomics study of the 4T1 breast cancer cell line, where cells were treated with MnP/ascorbate, Keap1 was 3.73-fold oxidized relative to nontreated cells [4, 80]. Three scenarios may possibly account for such data: (i) Nrf2 might have been regulated by a different mechanism other than by MnP-driven oxidation of Keap1 (summarized in reference [4]), or (ii) activation of this pathway by MnP could have lessened the anticancer effect of MnP; or, most likely, (iii) Nrf2 might have been activated, and endogenous antioxidative defenses subsequently upregulated, but oxidized by the action of MnP/H_2_O_2_ (as found by redox proteomics and detailed above). In turn, those antioxidants would be inactive and could not protect tumor from excessive oxidative stress and therefore cannot allow for its proliferation.

Finally, it may be obvious, but still important to note, that if the normal cell is under immense oxidative stress approaching the state of inflammation similar to that of a cancer, Mn porphyrins would have plenty of H_2_O_2_ to catalyze the oxidation of protein thiols, inactivate numerous proteins, and in turn aggravate the existing condition, rather than heal the diseased cell. We have reported such cases in a study on advanced diabetes and excessive radiation-induced rectum injury of a rat [110, 111]. In the former case, in a streptozotocin mouse model of diabetes, the methyl analog, MnTM-2-PyP^5+^ protected kidneys when the treatment started at the onset of diabetes, but induced kidney damage when the treatment started once diabetes already progressed [111]. In the latter case, when the rectum of rats was exposed to 20-30 Gy of radiation, MnTE-2-PyP^5+^ was protective and reduced the fraction of rats with proctitis. However, at doses higher than 30 Gy, Mn porphyrin amplified the cytotoxic effect of radiation [110].

## 6. MnTnBu-2-PyP^5+^, a Clinical Candidate, as a Cancer Radio- and Chemosensitizer while a Protector of Normal Tissue

**Figure 3:**
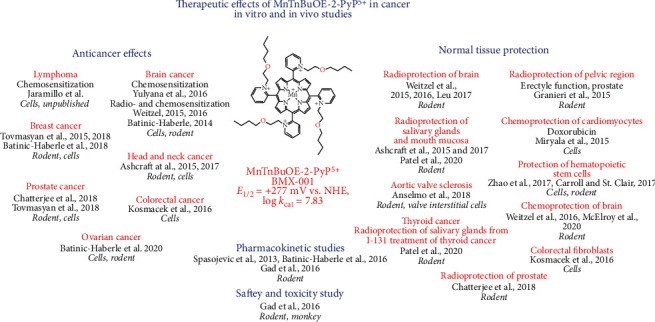
Therapeutic effects of MnTnBuOE-2-PyP^5+^ in suppressing cancer and protecting normal tissue [3, 4, 12, 24, 43, 54, 56, 58, 60, 82, 93, 100, 101, 114–122]. Such effects are driving progress of MnTnBuOE-2-PyP^5+^ into clinical trials. We have also listed here those beneficial effects upon normal tissue that are of relevance to cancer therapy. Such is the effect of MnTnBuOE-2-PyP^5+^ on suppression of aortic sclerosis/stenosis in cellular and mouse models, *via* reducing oxidative stress [123]. The effect is relevant to cancer patients subjected to chemotherapy as it increases the likelihood of plagues in their vasculature. Radiation of cancer patients often damages bone marrow, a source of hematopoietic stem/progenitor cells; the ability of MnTnBuOE-2-PyP^5+^ to enhance the number and function of those cells was reported [82]. Indeed, related to the ability of MnP to protect bone marrow, it was observed that none of the nine patients receiving MnTnBuOE-2-PyP^5+^/temozolomide in a Phase I glioma clinical trial developed dose-limiting thrombocytopenia [14].

Due to its progression into clinical trials, we have herein summarized primarily the therapeutic effects of MnTnBuOE-2-PyP^5+^ (Figure 3). Therapeutic effects of other MnPs were reviewed elsewhere [3, 25, 28, 68, 69, 74]. In cancer cells relative to normal cells, H_2_O_2_ metabolism is perturbed, resulting in elevated levels of H_2_O_2_. Either the production of H_2_O_2_ is increased or the enzymatic systems that should have maintained the physiological levels of H_2_O_2_ are downregulated or excessively oxidized/*S*-glutathionylated and thus are no longer active [3, 4, 74]. In turn, cancer is under oxidative stress and vulnerable to any further increase in it. It is thus no wonder that tumors were treated with therapies (such as radiation, chemotherapy, or ascorbate) that increase their oxidative stress and plunge them into apoptosis [27]. We and others have applied such therapies where different MnPs were combined with one or all of those treatments in cellular and animal models of breast and ovarian cancer, glioblastoma multiforme, head and neck cancer, hematologic malignancies, prostate cancer, and colorectal cancer. Anticancer effects were demonstrated, while normal tissue was protected by MnPs, see also under “Molecular Pathways Affected by Mn Porphyrins in Cancer and Normal Cell” [3, 68, 112, 113].

**Figure 4:**
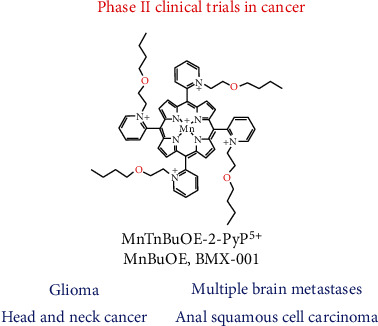
MnTnBuOE-2-PyP^5+^ (BMX-001) in four Phase II clinical trials. MnTnBuOE-2-PyP^5+^ was shown safe and well tolerated in a Phase I trial on glioma patients [13] and was then forwarded into Phase II trials. Initial data from the Phase I trial suggest the therapeutic potential of MnTnBuOE-2-PyP^5+^ with glioma patients [14, 15]. None of the nine patients receiving MnTnBuOE-2-PyP^5+^/temozolomide developed dose-limiting thrombocytopenia in the Phase I clinical trial [14]. Three other trials are ongoing on the radioprotection of normal tissue with head and neck cancer patients, anal squamous cell carcinoma patients, and patients with multiple brain metastases.

MnTnBuOE-2-PyP^5+^was found safe and well-tolerated in Phase I clinical trial on glioma [13] and is now in four Phase II clinical trials with the primary goal of demonstrating the radioprotection of normal tissues with cancer patients (Figure 4). Its efficacy is being tested in Phase II trials on the radioprotection of normal tissue with glioma patients (in combination with radiation and temozolomide, NCT02655601), head and neck cancer patients (in combination with radiation and cisplatin, NCT02990468), and anal squamous cell carcinoma patients (in combination with 5-fluorouracil/mitomycin, NCT03386500), as well as on the radioprotection of normal brain in cancer patients who suffer from brain metastases (in combination with radiation, NCT03608020). It is important to note that BMX-001 does not protect cancer cells or cancerous tissues but acts as a tumor radio- and chemosensitizer [3, 4]. A second porphyrin analog, MnTE-2-PyP^5+^ (AEOL10113, BMX-010), is being tested in a Phase II trial for a noncancer application—atopic dermatitis and itch (NCT03381625).

## 7. Ascorbate Enhances the Anticancer Radio- and Chemosensitizing Effects of MnTnBuOE-2-PyP^5+^

The treatment with MnP/ascorbate was thus far only investigated in cellular and mouse models of breast and ovarian cancers in combination with chemo- and radiotherapy. Herein, we are providing the overview of the reported data on MTE-2-PyP^5+^ and MnTnBuOE-2-PyP^5+^ in the 4T1 breast cancer cell line and MnTnBuOE-2-PyP^5+^ in CAOV2 and HOC7 ovarian cancer cell lines. It is noteworthy that MnP/ascorbate is not toxic to normal cells such as the human epithelial breast cell line, HBL-100 [12]; normal human astrocytes, iNHA; primary normal human dermal fibroblasts, NHDF; and the nontumorigenic epithelial breast cell line, MCF-10A [3, 28].

### 7.1. Breast Cancer

The impact of MnPs (combined with different sources of H_2_O_2_) on signaling pathways involved in the 4T1 mouse breast cancer cell line [4, 12, 43, 91], inflammatory aggressive breast cancer SUM149 and SUM190 cell lines [92], and MCF-7 and MDA-MB-231 metastatic breast cancer cell lines [98] were extensively studied. Data are summarized under “Molecular Pathways Affected by Mn Porphyrins in Normal and Cancer Cell” and Figure 2. In 4T1 mouse breast cancer cells and inflammatory breast cancer cells, NF-*κ*B and MAPK signaling pathways were the major ones affected by MnP when combined with radiation- and ascorbate-derived sources of H_2_O_2_ [4, 80]. The impact of MnPs on the Nrf2 pathway in cancer cells has not yet been reported.

We have most frequently utilized a 4T1, triple negative, metastatic mouse breast cancer cell line for therapeutic and mechanistic purposes in cellular studies and in the immunocompetent balb/c mouse. We have tested a variety of Mn porphyrins (MnTE-2-PyP^5+^, MnTnHex-2-PyP^5+^, MnTnBuOE-2-PyP^5+^, MnTFE-2-PyP^5+^, and MnTF_3_Pen-2-PyP^5+^), in combination with ascorbate and radiation (RT), on the impact of their different redox and bioavailability properties on anticancer efficacies [4, 12, 23, 43]. The chemistry behind such efficacies is elaborated under “Reactivity of Mn Porphyrins towards Protein Cysteines -Thiol Signaling,” and briefly summarized below.

**Figure 5:**
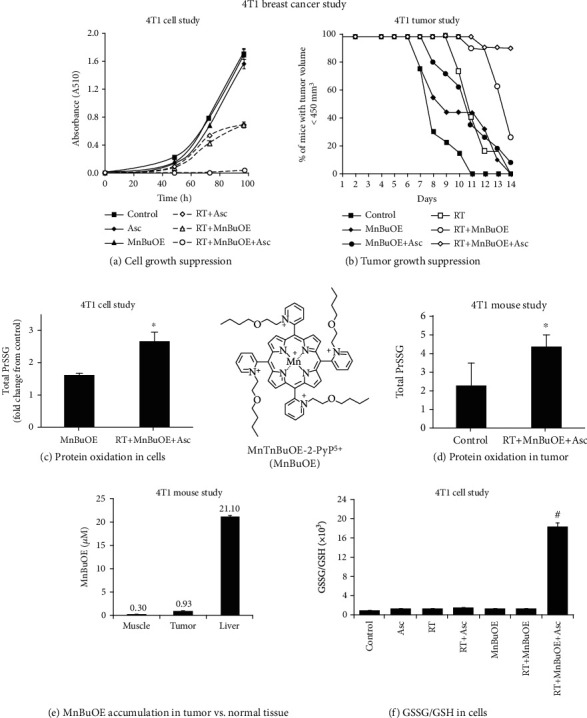
Ascorbate enhances the radiosensitizing ability of MnTnBuOE-2-PyP^5+^ to suppress cancer growth. *In a cellular study* (a), 4T1 cells were treated with 1 mM ascorbate (Asc) and 2.5 *μ*M MnTnBuOE-2-PyP^5+^ (MnBuOE). The 5 Gy radiation (RT) was delivered to cells at 1.81 Gy/min, and cell growth was followed during 96 h. Cell proliferation was assessed by sulforhodamine B assay, and measured as absorbance at 510 nm. Experiments were done in triplicate. Values are mean ± SEM. *In a mouse study*, 10^6^ tumor cells in 100 *μ*L were subcutaneously (sc) injected into a mouse flank. MnP was given sc at 2 mg/kg/day to balb/c mice for the duration of the study. Ascorbate was injected intraperitoneally for 5 days at 4 mg/kg/day, followed by 1 g/kg/day for the rest of the study. Treatment with MnP and ascorbate started one day before RT. Radiation started once the mice reached a tumor volume of ~80 mm^3^. RT was given at 2.5 Gy/day for 3 days. The number of mice per group was 12 [43]. On day 11 (b), >90% of mice treated with MnBuOE/Asc/RT had tumors less than 450 mm^3^ in size. The impact of MnP on the cellular (c) and tumor (d) redox status was demonstrated with the highest levels of total oxidized/*S*-glutathionylated proteins (PrSSG) when it was combined with both radiation and ascorbate—the system that induces the highest production of H_2_O_2_. The accumulation of MnP in the tumor, relative to the muscle from the leg opposite to tumor-bearing leg and liver (which were both not exposed to radiation), was measured by LC-MS/MS (e). An increase in the oxidative cellular redox environment by the action of MnP/ascorbate/radiation was demonstrated by the increase in the ratio of (GSSG)/(GSH) in 4T1 cells (f).

MnTnBuOE-2-PyP^5+^ was reduced with ascorbate and reoxidized with oxygen in a catalytic cycle, thereby producing excessive levels of superoxide/H_2_O_2_. H_2_O_2_ levels were further increased by the addition of radiation (RT) (equations (1)–(3)). Subsequently, MnTnBuOE-2-PyP^5+^ reacts with H_2_O_2_ and is oxidized to highly oxidizing Mn(V) dioxo species (equation (4)). This species in turn oxidizes/*S*-glutathionylates cysteines of signaling proteins (equations (5)–(7)). The yield of oxidized/*S*-glutathionylated proteins is high (Figures 5(c) and 5(d)) when the levels of colocalized reactants, MnTnBuOE-2-PyP^5+^ (Figure 5(e)) and H_2_O_2_, are high, giving rise also to high levels of oxidized glutathione, GSSG (Figure 5(f)). The high level of protein oxidation results in the promotion of apoptotic processes [4, 12, 43]. Due to the role of Mn porphyrin as a catalyst, in 4T1 mouse breast cancer studies we were able to obtain the therapeutic effects of the same magnitude with 2 mg/kg/day and 0.2 mg/kg/day [4, 12]. Such findings are clinically relevant as the lower dose is similar to the dose used in clinical trials.

In initial 4T1 mouse breast cancer studies, we only looked at the anticancer effect of MnTE-2-PyP^5+^ in its own right. The significant suppression of tumor growth was only achieved when it was given subcutaneously at 15 mg/kg/day throughout the study, but not at 2 mg/kg/day. At that point in time, we did not fully comprehend the mechanism of the anticancer action of MnPs [124]. We now know that MnP couples with endogenous sources of H_2_O_2_, including the H_2_O_2_ produced by its cycling with endogenous ascorbate. Even with levels of MnP as high as 15 mg/kg/day, the endogenous H_2_O_2_ levels sufficed only for the modest anticancer effect of MnP [124]. We needed much higher levels of MnP as a single agent, to suppress tumor growth, than when it is combined with exogenous sources of H_2_O_2_. In a subsequent study, a larger anticancer effect was seen when MnP was administered at 0.2 or 2 mg/kg/day concurrent with intraperitoneal ascorbate at 4 g/kg/day [12]. The largest anticancer effect on cellular viability and tumor growth was seen when Mn porphyrin was combined with two sources of H_2_O_2_—ascorbate and radiation (Figures 5(a) and 5(b)) [43]. When combined with ascorbate, MnTnBuOE-2-PyP^5+^ reduced cellular viability dose-dependently by up to 98%, and tumor growth by 36% *vs.* control. Further, ascorbate enhanced the radiosensitizing ability of MnTnBuOE-2-PyP^5+^ to suppress the cell viability dose-dependently by up to 98%, and tumor growth by 100% *vs.* control [4, 12, 43]. MnTnBuOE-2-PyP^5+^ and ascorbate, at doses used in cytotoxicity experiments (Figure 5(a)), have shown no toxicity when applied alone, whereas their combination have shown statistically significant anticancer effects compared to their respective single treatment cohorts. Moreover, both MnTnBuOE-2-PyP^5+^ and ascorbate sensitize cancer cells towards RT. The results indicate possible synergism between the treatment components.

In cellular and mouse 4T1 breast cancer studies, we have also compared the effects of MnTE-2-PyP^5+^ and MnTnBuOE-2-PyP^5+^ to EUK-8, MnTBAP^3-^, and M40403. M40403 is the Mn(II) cyclic polyamine whose enantiomer, GC4419, is in a clinical trial for an identical application as MnTnBuOE-2-PyP^5+^ [4, 43]. Only MnTE-2-PyP^5+^ and MnTnBuOE-2-PyP^5+^ exhibited anticancer effects in cellular and mouse models (Figure 5) and restored the cellular physiological environment as measured *via* GSSG/GSH redox couple and levels of *S*-glutathionylated proteins (Figures 5(c), 5(d), and 5(f)) [43]. The origin of the effects with cationic Mn porphyrins and the lack of those with other compounds were discussed in detail elsewhere [4, 43]. Briefly, based on low catalase-like activities, EUK-8, MnTBAP^3-^, and M40403 have no ability to cycle with H_2_O_2_. Further, they have a low ability to oxidize ascorbate. Finally, all 3 compounds have low (MnTBAP^3-^, EUK-8) or no GPx-like activity (M40403). Those properties are essential when coupling with ascorbate and subsequently with H_2_O_2_ in order to give rise to high-valent Mn compounds that would in turn oxidize protein thiols.

### 7.2. Ovarian Cancer

Epithelial cancers of the ovary, fallopian tube, and peritoneum (collectively known as epithelial ovarian cancer cells (EOCs)) represent 85% to 90% of all ovarian cancers and are treated in a similar manner. The majority of EOCs are aggressive *high-grade serous cancers (HGSCs)* and are associated with a worse clinical outcome [125, 126]. Further, HGSCs are especially challenging due to their primary and secondary chemoresistance. HGSCs almost always harbor tumor protein TP53 mutation and lack obvious benign and/or borderline tumors, except in rare cases [127]. Recent data suggest that TP53 dysfunction in the fallopian tubal cells triggers high-grade serous carcinogenesis [128]. Also, *bcl-2* (a major regulator of apoptosis and tumor pathogenesis, progression, and resistance to treatment) was overexpressed in HGSC specimen from women with shorter overall survival of >3 years (*p* = 0.007) in comparison with women with a longer overall survival of >7 years [129]. Findings were confirmed using an external HGSC database, GSE26712 (Siamakpour-Reihani), showing that women with high *bcl-2* tumor expression had significantly worse survival (*p* = 0.002) and approximately 2-fold higher risk of death. The *bcl-2* gene was transcriptionally regulated by NF-*κ*B and directly links the tumor necrosis factor *α*, TNF-*α*/NF-*κ*B signaling pathway with bcl-2 expression and its prosurvival response in human prostate carcinoma cells [130]. DNase I footprinting, gel retardation, and supershift analysis identified an NF-*κ*B site in the bcl-2 p2 promoter [130]. Several studies in different cell types have shown that treatment with known activators of NF-*κ*B often results in an increase in prosurvival factors such as bcl-2, bcl-xL, and bfl-1/A1 [131–134]. Conversely, *low-grade serous cancers* (*LGSC*s) often intermingle with the benign and borderline serous tumor component and exhibit low cellular proliferation. LGSC and serous borderline tumors have relatively frequent point mutations in the *KRAS* and *BRAF* genes [135, 136] which participate in RAS-RAF-MEK-ERK signaling (also known as MAPK/ERK). Thus, the occurrence of these 2 mutational events in low-grade serous carcinogenesis is mutually exclusive [127]. Reportedly, mutations occur at codon 599 of *BRAF* and codons 12 and 13 of *KRAS* [136]. Either of those, but not both, are present in 15 out of 22 (68%) invasive low-grade micropapillary serous carcinomas (MPSC), and in 31 out of 51 (61%) serous borderline tumors, precursors to MPSC. Mutations are specific for LGSC and none were found in 72 conventional HGSCs [136].

**Figure 6:**
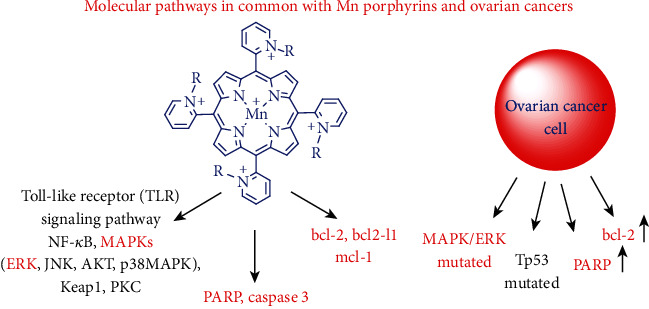
Molecular pathways upregulated in ovarian cancer cells that coincide with those which are affected by Mn(III) *N*-substituted pyridylporphyrins. Common pathways are indicated in red. In breast cancer studies, MnPs were found to operate predominantly *via* the TLR pathway with NF-*κ*B, p38 MAPK, MAPK14, and HSP60 being components of it. It was also found that MnP inhibits the antiapoptotic proteins, bcl-2 and mcl-1, which are overexpressed in high-grade serous cancer cells [129], thus promoting apoptotic pathways (see Figure 2). PARP is also overexpressed in several types of ovarian cancer cells, and MnP was found to suppress its activity *via* increasing levels of inactive cleaved PARP (Figure 2) [137]. MnP was also found to increase the levels of activated, cleaved caspase 3 [137]. TP53 and MAPK/ERK are mutated in low-grade serous cancer [127] allowing for the therapeutic interventions with their inhibitors, MnPs prospectively being one of those. R in the structure of MnPs indicates *ortho-N*-alkyl- or *N*-alkoxyalkylpyridyl substituents.

We have demonstrated that Mn(III) *N*-substituted pyridylporphyrins favorably affect pathways that are involved in LGSC or HGSC such as bcl-2/NF-*κ*B, and MAPK/ERK (see more under “Molecular Pathways Affected by Mn Porphyrins in Cancer and Normal Cells” and Figures 2 and 6) [3, 4, 74, 80, 91, 92, 94]. Such data have provided strong basis for exploring the therapeutic potential of MnPs in ovarian cancer.

**Figure 7:**
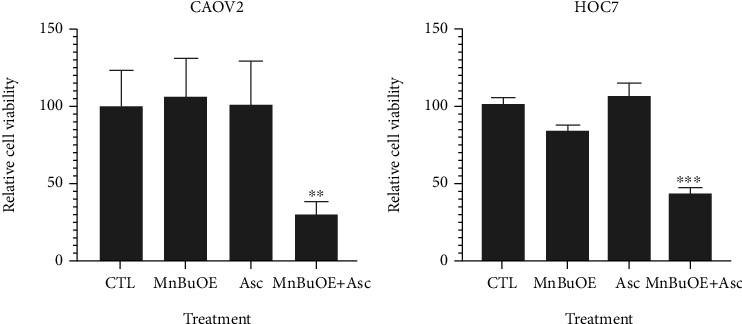
Ascorbate sensitizes high- (CAOV2) and low-grade (HOC7) ovarian cancer cells to MnTnBuOE-2-PyP^5+^. Cancer cells were seeded onto a 96-well plate with RPMI 1640 medium plus 10% fetal bovine serum and 1% penicillin and streptomycin. The drugs were added onto cells after 24 hours of plating; treatment was applied for 72 hours. The cell viability was assessed using CellTiter-Glo® Luminescent Cell Viability Assay (Promega) according to the manufacturer's protocol. Relative cell viability was calculated against mock treatment (CTL). The average values at each dose were calculated from 4 replicates and normalized against control values (CTL). *Statistics*: the paired Student *t*-test was performed. The*p*values are shown here as ^∗∗^*p* < 0.01, and ^∗∗∗^*p* < 0.001 relative to control. *Abbreviations*: MnBuOE: MnTnBuOE-2-PyP^5+^; Asc: ascorbate; CTL: control. *Concentrations of drugs for COAV2 cells*: MnBuOE = 2.5 *μ*M, Asc = 0.25 mM. *Concentrations of drugs for HOC7 cells*: MnBuOE = 5 *μ*M, Asc = 0.25 mM [138, 139].

The impact of MnTnBuOE-2-PyP^5+^, combined with ascorbate and chemotherapy, was explored in two ovarian cancer cell lines, each representative of low- (HOC7) and high-grade (CAOV2) carcinomas [138, 139]. Data show the impressive ability of MnTnBuOE-2-PyP^5+^ (MnBuOE)/ascorbate (Asc) to suppress the growth of both ovarian cancer cells (Figure 7) [138, 139]. Ascorbate sensitized CAOV2 and HOC7 ovarian cancer cells to MnTnBuOE-2-PyP^5+^, reducing cellular viability by 75% and 42%, respectively (Figure 7)[139]. The data pointed to the critical role that catalytic redox cycling of Mn porphyrin with ascorbate plays in MnP-driven tumor suppression giving rise to excess peroxide. The mouse tumor growth studies on CAOV2 cell line demonstrated the strong anticancer potential of MnTnBuOE-2-PyP^5+^/ascorbate [138]. Such results agree well with effects seen in 4T1 breast cancer cellular and mouse studies ([12, 43]. At least in part, such effects are orchestrated through common signaling pathways comprised of NF-*κ*B, bcl-2, MAPK/ERK, PARP, and caspase 3; see also under “Molecular Pathways Affected by Mn Porphyrins in Normal and Cancer Cell” (Figures 2 and 6) [3, 91, 92].

Paclitaxel and carboplatin are standard-of-care therapy for ovarian cancer [140]. However, paclitaxel and carboplatin are associated with significant toxicities that include grade 3-4 thrombocytopenia, grade 3-4 neutropenia, grade 2-4 neurotoxicity [140–142], and a decline in cognitive functions [143]. In a Phase I glioma clinical trial on the radioprotection of normal tissue with glioma patients (Figure 4), [13] none of the nine patients receiving MnTnBuOE-2-PyP^5+^/temozolomide developed dose-limiting thrombocytopenia [14], which is associated with chemotherapy in up to 39% of patients [140]. This effect of MnTnBuOE-2-PyP^5+^ is related to its ability to protect the bone marrow from radiation, and seems to be at least in part related to the MnP-driven inhibition of NF-*κ*B and activation of Nrf2 [82, 144, 145]. MnTnBuOE-2-PyP^5+^ was found to enhance the number and function of hematopoietic stem/progenitor cells isolated from the bone marrow *via* activation of the Nrf2 transcription factor and upregulation of its endogenous antioxidative genes (see also “Molecular Pathways Affected by Mn Porphyrins in Normal and Cancer Cell” and Figure 2**)** [82].

## 8. Future Perspectives

Mn porphyrins are now in clinical trials on several types of cancer: glioma, head and neck cancer, anal squamous cell carcinoma, and multiple brain metastases emerging from different primary cancers. We have summarized here the therapeutic effects of MnPs, and in particular, the anticancer efficacy of the MnP/ascorbate system. Thus far, we explored such system in the treatment of breast and ovarian cancer in preclinical models. In a breast cancer study, ascorbate further amplified the anticancer effect of MnP/radiation. In the treatment of ovarian cancer, data suggest that the MnP/ascorbate system may be an anticancer agent as powerful as paclitaxel—a standard-of-care therapy.

Ascorbate was used in preclinical models in combination with chemo- and radiotherapy for several different cancers [113, 146–148]. Ascorbate has reached clinical trials as an adjuvant to standard-of-care therapy in a number of cancers such as glioma, pancreatic cancer, prostate cancer, kidney cancer, hematologic malignancies, bone sarcomas, and non-small-cell lung cancer [113, 149]. It was combined with temozolomide and radiotherapy in a Phase I clinical trial of newly diagnosed glioblastoma; the system was found safe and with promising clinical outcome and warranted further investigation in an ongoing Phase II trial (NCT02344355) [150]. Ascorbate is in progress in a Phase II trial (NCT02905578) for the treatment of pancreatic cancer in combination with nab-paclitaxel and gemcitabine standard-of-care therapy [151, 152]. Thus far, only one pilot Phase I/IIa trial on ascorbate in ovarian cancer was conducted on patients diagnosed with stage III or IV ovarian cancer. 27 patients were randomized into either a paclitaxel/carboplatin group or a paclitaxel/carboplatin/ascorbate group [153]. Treatment with ascorbate reduced chemotherapy-related toxicity. There was an 8.75-month increase in progression-free survival and an improved trend in overall survival in the ascorbate-treated arm. Ascorbate is now in a Phase I clinical trial for the treatment of pneumonia with COVID-19 patients (NCT04264533).

The mechanism of action of ascorbate (in the absence of MnPs) is reportedly based on its coupling with metal centers of endogenous proteins/enzymes, giving rise to H_2_O_2_ [146, 147, 154]. Yet, those metalloproteins are optimized neither on kinetic nor thermodynamic grounds for reaction with ascorbate. The MnPs are, however, optimized to couple with ascorbate. In a catalytic fashion, MnPs produce large amounts of H_2_O_2_ which can then be employed (again in a catalytic fashion) to oxidize and inactivate cellular proteins. We thus hope that the MnP/ascorbate system will eventually progress into clinical trials.

For the first time in drug development, redox-active metal complexes that affect cellular redox environment, but do not act on a particular biomolecule, are in clinical trials: two Mn porphyrins (MnTnBuOE-2-PyP^5+^, BMX-001 and MnTE-2-PyP^5+^, BMX-010) and Mn cyclic polyamine (GC4419). Moreover, BMX-001 and GC4419 are presently tested for the same clinical application: radioprotection of the normal tissue with head and neck cancer patients. (NCT03689712). The results from the Phase II trial on GC4419 were reported [155], indicating significant, clinically meaningful reduction in severe oral mucositis duration, incidence and severity with acceptable safety at dose of 90 mg [155]. The Phase III trial on GC4419 has begun (NCT03689712). The outcome of the trial could advance our understanding of the role redox chemistry has in cellular biology under pathological and physiological conditions and further clinical development of the new class of therapeutics. While BMX-001 and GC4419 are similarly potent SOD mimics, these compounds have a different chemistry and therefore may undergo different reactions in the cellular environment. With their Mn center in +2 oxidation state, Mn(II) cyclic polyamines are not very stable complexes and are prone to demetallation, whereas Mn(III) porphyrins with their Mn in +3 oxidation state are stable compounds. Indeed, Weekley et al. demonstrated that the enantiomer of GC4419, M40403, undergoes demetallation *in vivo* [35]. Such difference in the oxidation state of the Mn center also controls their reactivities. Data show that rather than acting as SOD mimics in both normal ant tumor cell/tissue, Mn porphyrins cycle with ascorbate, and at the expense of oxygen, give rise to H_2_O_2_. In a subsequent step, H_2_O_2_ is employed by Mn porphyrin in a catalysis of oxidation of protein cysteines. Such oxidative modification of proteins modifies their activities and affect cellular metabolism. The reactivity of GC4419 towards ascorbate was reported, but its mechanism was not readily understood [156]. The Mn +2 state of GC4419 reacts with O_2_^•-^ in a 1st step to give rise to the Mn +3 state (and H_2_O_2_) and cycles back to Mn +2 either with O_2_^•-^ or with ascorbate, whereby either oxygen or ascorbyl radical, respectively, are formed. The Mn +2 state is a resting state of GC4419 and is not expected to react with oxygen in a reaction which would give rise to O_2_^•-^ and subsequently H_2_O_2_, and thus enable the anticancer action of GC4419 *via* its high-valent oxidation state capable of oxidizing protein thiols. The reaction of the Mn +3 state of GC4419 with ascorbate instead of with O_2_^•-^, to close the catalytic cycle, may enhance the reactivity of GC4419 as an SOD mimic. Yet, the yield of H_2_O_2_ (and the Mn +3 state) in the 1st step, to allow for the 2nd step reaction of Mn +3 with ascorbate, is limited by the levels of superoxide. For the same reason, the oxidation of GC4419 with H_2_O_2_ to a high-valent Mn +4 oxidation state would also occur with a low yield; moreover, such reaction would likely give rise to the degradation of the complex [35]. The yield of H_2_O_2_ in the case of MnPs, however, is controlled by the amount of oxygen and ascorbate, both of which are abundant.

Our studies have demonstrated that M40403, an enantiomer of GC4419, has no GPx-like activity [4, 157], low activity towards ascorbate [43] and H_2_O_2_ [104], and has no anticancer efficacy in the presence of ascorbate in suppressing tumor growth in 4T1 mouse breast cancer model [4, 43]. Based on our present knowledge, the ability of MnPs to protect normal tissue relates to their ability to catalyze the oxidative modifications of signaling proteins such as NF-*κ*B and Keap1 of Nrf2. Yet, the mechanism behind the protective actions of GC4419 is not fully understood. Therefore, more studies are needed to clarify the mechanism of anticancer actions of Mn(II) cyclic polyamines in the presence and absence of ascorbate.

With regard to the use of Mn porphyrin/ascorbate treatment for the protection of the normal tissue, the concern may be whether the H_2_O_2_ originating from MnP/ascorbate cycling will induce cytotoxicity. We have performed a study with MnTF_3_Pen-2-PyP^5+^ on the radioprotection of the prostate and erectile function in the presence of ascorbate. No toxicity to normal tissue was seen [24]. Lack of the toxicity arises from the ability of normal tissue to maintain H_2_O_2_ at the physiological levels due to the abundant and functional endogenous enzymes and small-molecular-weight antioxidants (see “Molecular Pathways Affected by Mn Porphyrins in Cancer and Normal Cell”). Yet, more studies on different animal models are needed for further evaluation of such system in the radioprotection of normal tissue.

While we have fully characterized the aqueous chemistry of two members of the newest class of Mn(III) fluorinated *N*-alkylpyridylporphyrins, a lot is left to be done on their *in vitro* and *in vivo* therapeutic effects. Thus far, we demonstrated that they are anticancer drugs at least as good as MnTE-2-PyP^5+^ and MnTnBuOE-2-PyP^5+^in a 4T1 mouse breast cancer model in the presence of radiation and ascorbate [23]. Data also show that, in the presence of ascorbate, they possess the ability to radioprotect normal tissue [24]. The efficacy in other cancer cell lines and other inflammatory diseases are yet to be performed.

Given the rich redox chemistry of MnPs and the redox biology of a cell, much is left to be explored. On the level of a mechanism of action, the question that is still left unanswered is how MnPs affect Nrf2 regulation in a tumor. Nrf2 was activated by MnP in hematopoietic stem/progenitor cells [82]. Activation of Nrf2 was followed by the upregulation of MnSOD, catalase, glutathione-*S*-transferase p1, NAD(P)H quinone dehydrogenase 1, and uncoupling proteins. Redox proteomics of 4T1 breast cancer cells treated with MnP and ascorbate (as exogenous source of H_2_O_2_) showed that Keap1 is oxidized, thus Nrf2 is likely activated. The subsequent upregulation of antioxidants should have promoted tumor growth, but such impact on tumor growth by MnPs has not been reported [3, 4]. A possible explanation lies in the fact that MnP, combined with sources of H_2_O_2_, oxidizes antioxidant enzymes. Thus, despite their upregulation by the activated Nrf2, such antioxidants would likely be inactivated and thus would not diminish the anticancer potential of MnP.

## 9. Conclusions

Stable and redox-active Mn porphyrins were thus far developed, all of which carry positive charges on each of four *ortho* pyridyl nitrogens. Those charges provide thermodynamic and kinetic facilitation for the reactions of MnPs with biomolecules. MnPs were initially developed as powerful SOD mimics with the *k*_cat_ for the catalysis of O_2_^•-^ dismutation as high as 7 × 10^8^ M^−1^ s^−1^. Yet, their rich redox chemistry, originating from 4 biologically assessable oxidation states, +2, +3, +4, and +5, enables MnPs to couple with numerous components of the rich redox biology of the cell. Many more reactions of MnPs might be envisioned to emerge in the future.

Driven by the pentacationic charge, lipophilic *N*-alkylpyridyl substituents, and abundant anionic phosphates of the cell, MnPs distribute in various cellular organelles, mitochondria and nucleus included. MnPs distribute in all organs, including the brain and brain compartments. Finally, MnPs prefer tumor to normal cell/tissue accumulation.

On a therapeutic level, MnPs were found to act as anti-inflammatory drugs. Recently, most of the studies were concentrated on their efficacy as anticancer drugs, in combination with chemo- and radiation therapy, and as radioprotectors of normal tissue with cancer patients. The wealth of effects *in vitro* and *in vivo* has driven the progress of MnPs into clinical trials.

While direct reactivity with O_2_^•-^ cannot be fully excluded as their mechanism of action, numerous *in vitro* and *in vivo* studies suggest that therapeutic effects in both normal and tumor cells/tissues arise from the ability of MnPs to modify activities of cellular signaling proteins and thus affect proliferative and apoptotic pathways. Such studies, along with redox proteomics, helped us understand the mechanism below the ability of MnPs to affect signaling proteins. In the presence of H_2_O_2_ and glutathione, MnPs catalytically *S*-glutathionylate cysteines of numerous endogenous proteins, such as NF-*κ*B, Keap1, and MAPKs, and endogenous antioxidative defenses such as peroxiredoxins, glutathione-*S*-transferase, and isocitrate dehydrogenase. Such oxidative modifications result in the modification of the activities of proteins. The yield of oxidative modifications of proteins would depend upon the levels of MnPs and H_2_O_2_, both of which are at much higher levels in tumors than in normal tissues. Consequently, massive oxidation of tumor biomolecules would promote apoptotic processes. Conversely, lesser inhibition of NF-*κ*B and activation of Nrf2 (upon oxidation of Keap1) would restore the physiological environment of diseased but normal cell. The impact of MnPs on both NF-*κ*B and Nrf2 was investigated in normal cells/tissues, but only the effect on NF-*κ*B was thus far reported in tumors.

In summary, the ability of MnPs to modulate activities of major signaling proteins, along with high cellular, mitochondrial, and tissue bioavailability and stability, resulted in favorable therapeutic effects, which enabled their progress into several Phase II clinical trials.

## Data Availability

This is a review manuscript and all the data that are described here are from published material and can be found under the list of references.
